# Supplementary material for: DMRforPairs: identifying Differentially Methylated Regions between unique samples using array based methylation profiles
Source: BMC Bioinformatics. 2014 May 15;15:141. doi: 10.1186/1471-2105-15-141 (PMC4046028; doi:10.1186/1471-2105-15-141)
Supplement: Additional file 2 — DMRforPairs output for the comparison of A431-MCF7 and NA17018-NA17105. Please start from the HTML files in each folder. Available via the BMC Bioinformatics website. [file 1471-2105-15-141-S2.zip › 1394847754114233_MOESM2_ESM/A431_MCF7/figures/33802.pdf]

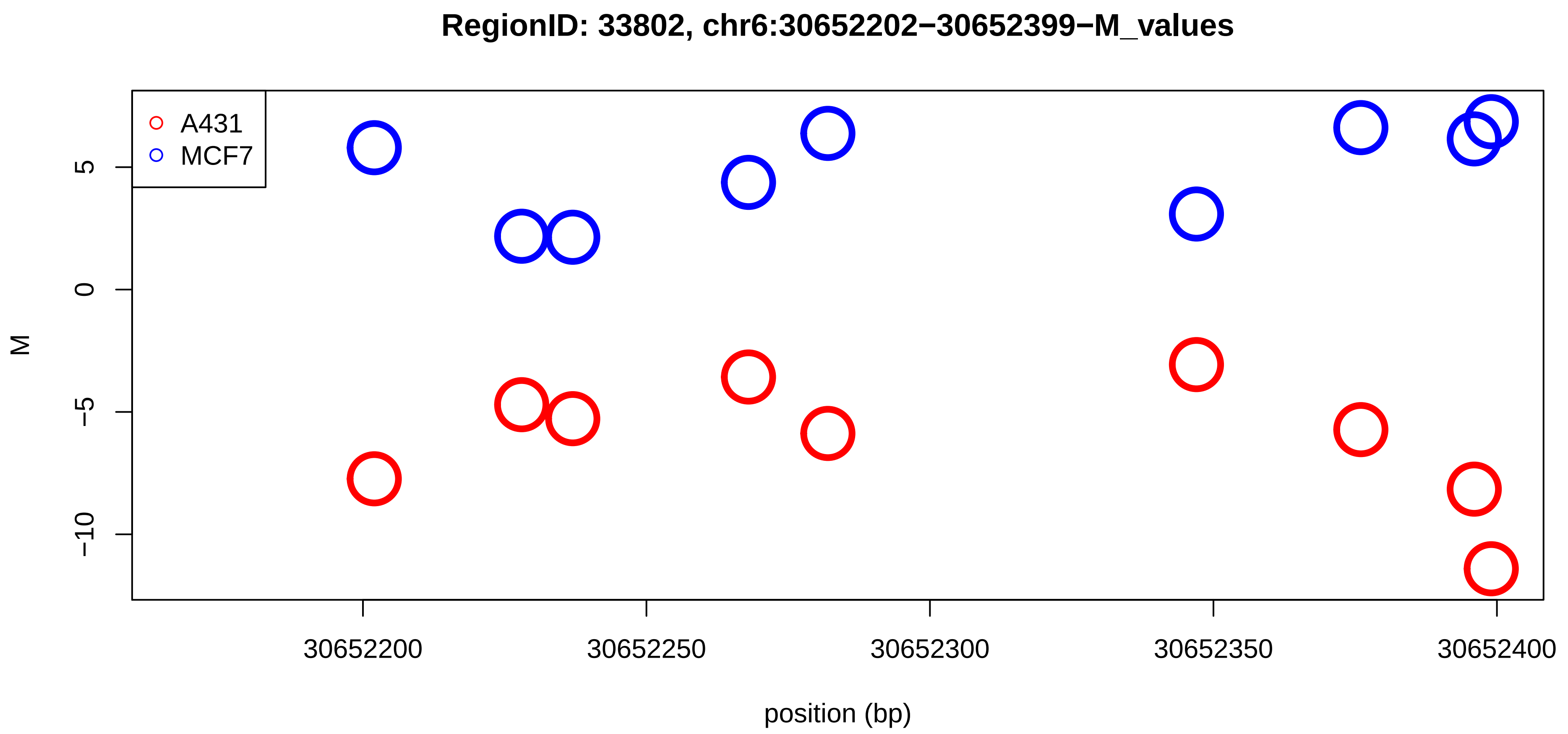
RegionID: 33802, chr6:30652202−30652399−M_values
A431
MCF7
M
5
0
−5
−10
30652200
30652250
30652300
30652350
30652400
position (bp)

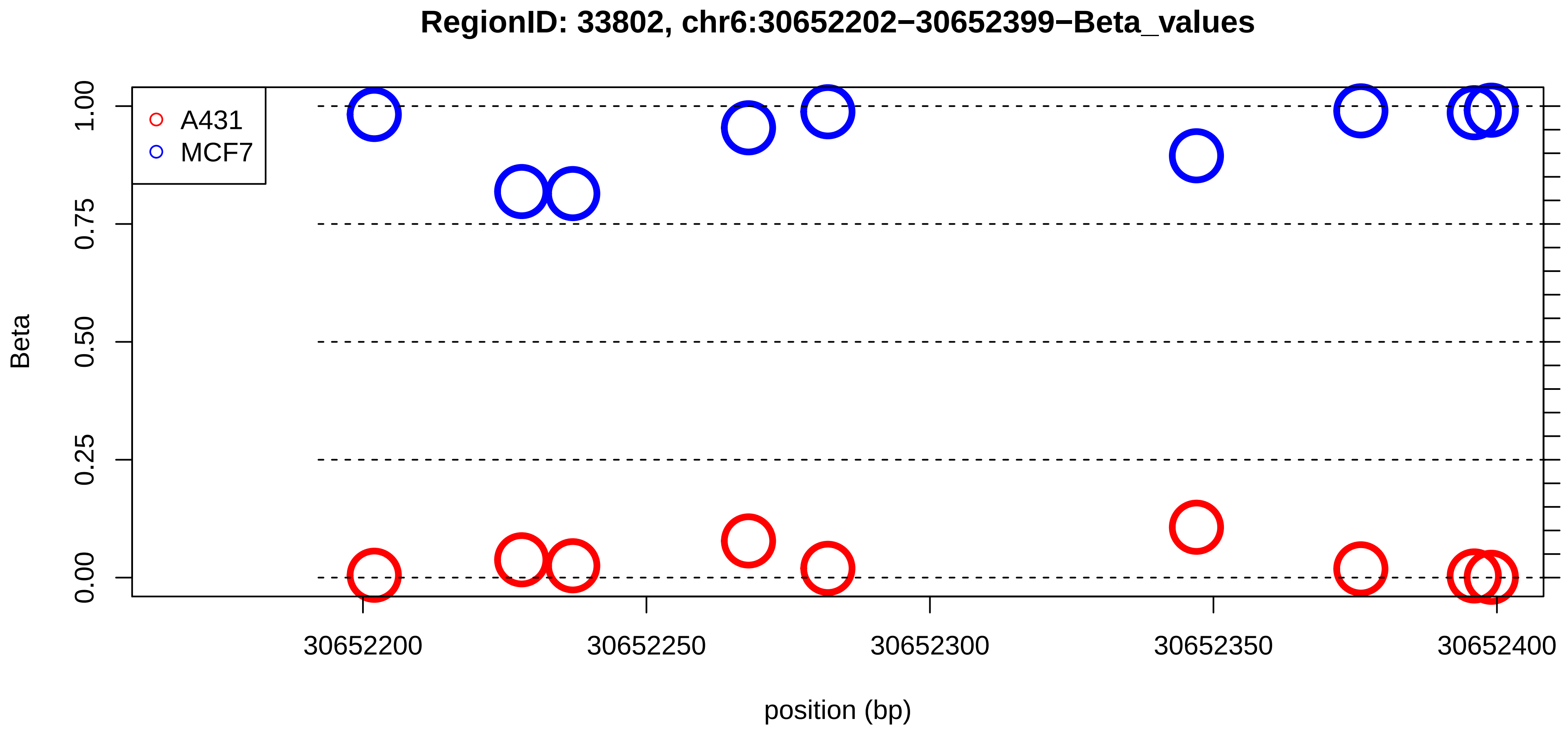
RegionID: 33802, chr6:30652202−30652399−Beta_values
A431
MCF7
Beta
1.00
0.75
0.50
0.25
0.00
30652200
30652250
30652300
30652350
30652400
position (bp)

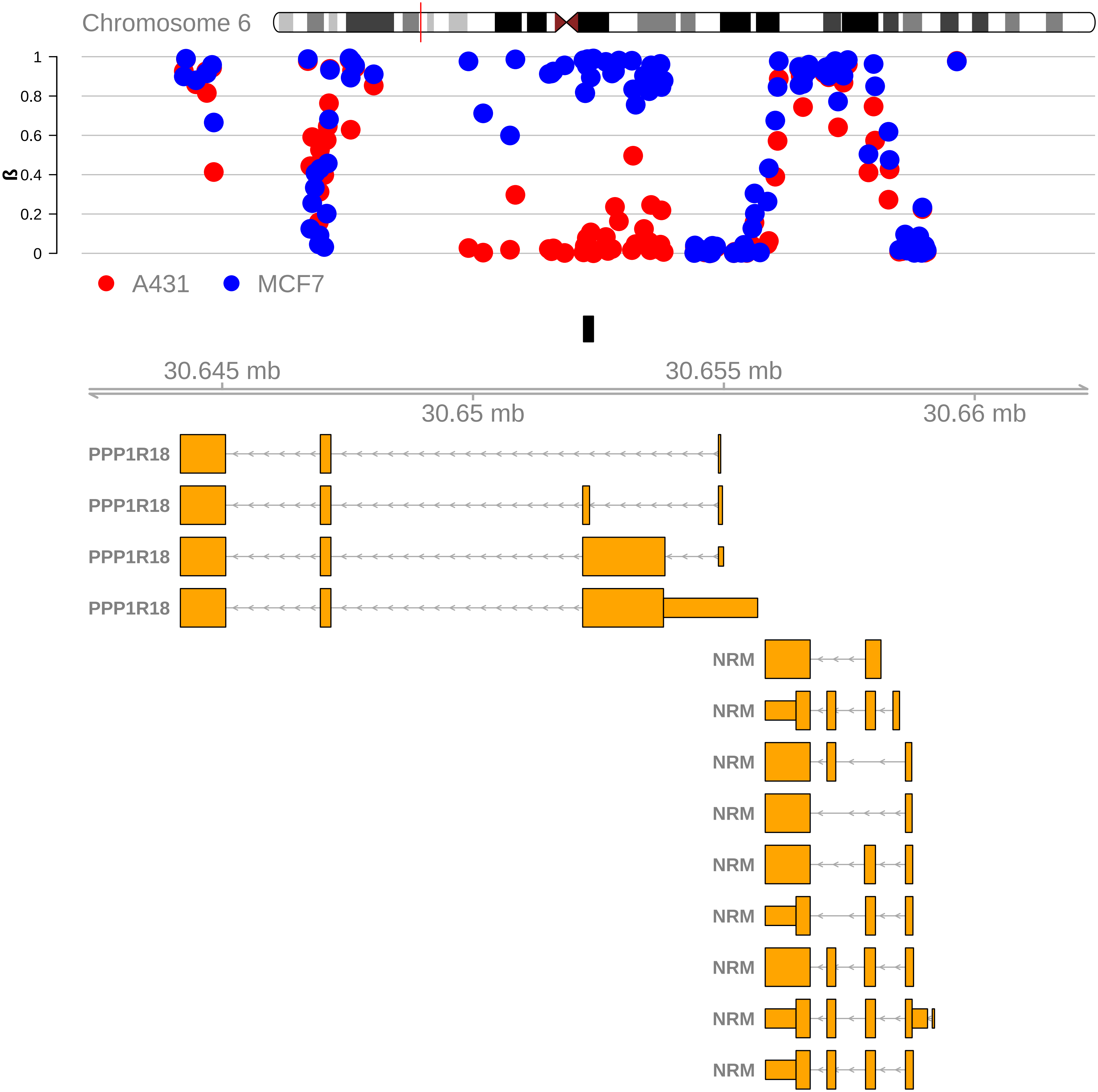

Chromosome 6
ß
1
0.8
0.6
0.4
0.2
0
A431
MCF7
30.645 mb
30.655 mb
30.65 mb
30.66 mb
PPP1R18
PPP1R18
PPP1R18
PPP1R18
NRM
NRM
NRM
NRM
NRM
NRM
NRM
NRM
NRM
